# The evolution of chemotherapy in brain tumors: from historical milestones to precision medicine in glioblastoma

**DOI:** 10.37349/etat.2026.1002374

**Published:** 2026-05-21

**Authors:** Alessia Ciafarone, Paola Palumbo

**Affiliations:** University of Saskatchewan, Canada; ^1^Department of Physical and Chemical Sciences, University of L’Aquila, 67100 L’Aquila, Italy; ^2^Department of Life, Health & Environmental Sciences, University of L’Aquila, 67100 L’Aquila, Italy

**Keywords:** glioblastoma, chemoresistance, temozolomide, tumor microenvironment, precision medicine

## Abstract

Chemotherapy has profoundly shaped modern oncology, evolving from early cytotoxic approaches to biologically informed strategies. In glioblastoma (GBM), the highly aggressive primary brain tumor, precision medicine does not rely on the identification of a single crucial oncogenic driver but integrates biologically informed stratification strategies to predict treatment response, resistance, and therapy-induced adaptation. This review recapitulates the historical milestones of chemotherapeutic development in neuro-oncology, with particular emphasis on GBM. As the main chemotherapeutic agent currently used in GBM, temozolomide (TMZ) initially represented a major therapeutic breakthrough; however, its clinical use has progressively unveiled the biological and clinical limitations of conventional cytotoxic paradigms. While TMZ exerts antitumor activity through DNA damage–induced apoptosis, accumulating experimental evidence indicates that it may also elicit adaptive responses, ultimately supporting tumor progression and therapy resistance. By integrating historical milestones with recent molecular understanding, this review highlights how improved knowledge of therapy-induced adaptations may inform emerging precision medicine strategies in GBM, underscoring the need for tailored treatments to overcome tumor heterogeneity and adaptive responses.

## Introduction

Malignant gliomas represent the most aggressive form of primary brain tumors, with glioblastoma (GBM) identified as the most common and aggressive subtype. These tumors are characterized by rapid growth and a marked ability to infiltrate surrounding brain tissue, making treatment extremely challenging. Historically, GBM was classified according to histopathological features, as defined by the World Health Organization (WHO) [1]. However, this conventional classification failed to recapitulate the biological and molecular GBM features. Over the last decade, genomic and transcriptomic profiling have deeply transformed the understanding of GBM, leading to the identification of distinct molecular subtypes such as classical, mesenchymal, proneural and neural, exhibit varying responses to standard treatments, like temozolomide (TMZ), the first-line chemotherapeutic agent [2]. The 2016 WHO classification introduced an integrated diagnostic framework for gliomas, combining histopathological evaluation with molecular markers such as the enzyme isocitrate dehydrogenase (IDH) mutation and 1p/19q codeletion [3]. The 2021 WHO classification (5th edition) further expanded this approach by emphasizing molecular profiling as crucial to central nervous system tumor classification. According to this classification, GBM refers specifically to IDH-wildtype diffuse astrocytic tumors classified as CNS WHO grade 4. In addition to histological features, these tumors can be defined by key molecular alterations, including TERT promoter mutation, EGFR amplification, and the combined gain of chromosome 7 with loss of chromosome 10 (+7/−10) [4]. The use of advanced techniques such as Next-Generation Sequencing (NGS) and DNA methylation profiling enables more precise tumor characterization and supports advancement in clinical strategy [4].

Despite integrated treatment strategies, the very poor outcome of GBM could be largely attributed to the high degree of intratumoral heterogeneity, complex interactions with the tumor microenvironment (TME), and the fast development of therapy resistance. In the context of GBM, resistance to TMZ is multifactorial. It involves DNA repair mechanisms such as O6-methylguanine-DNA methyltransferase (MGMT), a DNA repair enzyme that removes TMZ-induced O6-methylguanine lesions. The methylation status of the MGMT promoter is a well-established predictor of TMZ response. Additional mechanisms include altered drug metabolism and evasion of cell death pathways [5]. Beyond MGMT-mediated resistance, additional mechanisms contribute to TMZ resistance, including alterations in DNA mismatch repair pathways, overexpression of drug efflux transporters, and the presence of cancer stem cells exhibiting inherent resistance to chemotherapy [6, 7]. Clinically, many GBM patients, particularly elderly individuals, show limited responsiveness to TMZ therapy, which has been associated with DNA repair mechanisms such as MGMT activity and complex interactions with the immune system [8, 9]. Furthermore, the TME plays a pivotal role in modulating therapeutic efficacy [10].

## The historical evolution of diagnostics and therapy in brain tumors

Over the past century, key discoveries and therapeutic advances have progressively shaped the current understanding and management of malignant gliomas. The term “glioma” is derived from “glia”, the non-neuronal cells in the nervous system providing support and nutrition for neurons, playing crucial roles in maintaining homeostasis, forming myelin, and participating in signal transmission within the nervous system. In the mid-19th century, Rudolf Virchow, a pioneering German pathologist, first described glial cells laying the foundations of modern glioma pathology [11]. In 1926, Bailey and Cushing introduced the first histological classification of gliomas according to cellular characteristics [12]. Differentiating gliomas into subtypes, including astrocytomas, oligodendrogliomas, and ependymomas, this classification system was crucial in advancing the understanding of tumor behavior and tailoring therapeutic strategies.

Surgery has been, and continues to be, the primary approach in brain tumour management as it removes a large part of cancer cells and enhances the effectiveness of subsequent treatments. In 1884, one of the earliest successful brain tumor removals was performed by Victor Horsley, and on November 25 of the same year, Rickman J. Godlee conducted the first recognized resection of a primary brain tumor [13, 14]. However, early neurosurgical procedures were not free from high mortality due to limited anesthesia, lack of antibiotics and inadequate understanding of neuroanatomy. Later, in 1957, Theodore Kurze at the University of Southern California revolutionized neurosurgery, introducing the operating microscope into neurosurgical practice, significantly improving surgical outcomes and reducing postoperative complications [15]. Today, maximal safe tumor resection remains a cornerstone of GBM management and is associated with improved survival outcomes in patients with newly diagnosed GBM [16].

Importantly, the introduction of radiographic techniques (X-rays) marked a crucial advancement in the diagnosis of brain tumors, enabling tumors visualization in living patients. Before X-rays, diagnosis of brain tumors was based primarily on clinical symptoms and post-mortem examinations. The first documented use of X-rays in neurological conditions, including brain tumors, followed the discovery of X-rays by Wilhelm Röntgen in 1895 [17]. Although early radiography had limited sensitivity for gliomas due to poor tissue contrast and technical constraints, it represented the foundation of modern neuroimaging. Subsequent developments, including pneumoencephalography and cerebral angiography, progressively improved brain tumor localization and diagnostic accuracy [18]. Later, in the 1970s, the computed tomography (CT) scans, based on computer-processed X-rays, allowed for the creation of detailed tomographic images of the brain [19, 20]. Over time, X-ray technology advanced, eventually leading to the development of more sophisticated imaging techniques, such as Magnetic Resonance Imaging (MRI). The first MRI image was published in 1973 by the Nobel Prize Paul Lauterbur [21]. By 1981, MRI began to be used for imaging brain structures. This technology has revolutionized the resolution of glioma and other brain tumors identification, allowing for precise localization, accurate tumor volume definition, and in-depth assessment of infiltrative behavior and compressive effects on surrounding structures, along with critical insights into related complications [22]. Even today, MRI has become the gold standard for neuro-oncological imaging and represents the standard approach for the diagnosis and follow-up of gliomas. Conventional MRI sequences, including contrast enhanced T1 and T2/FLAIR imaging, are routinely used to evaluate tumor location, extent, and enhancement patterns [23, 24]. Advanced MRI techniques, such as diffusion-weighted imaging (DWI), perfusion-weighted imaging (PWI) and MR spectroscopy, provide additional information on tumor cellularity, vascular and metabolic features, thereby supporting characterization, differential diagnosis, and treatment monitoring [25]. In addition to MRI, positron emission tomography (PET) imaging has increasingly contributed to the evaluation of gliomas, particularly GBM. PET tracers targeting tumor metabolism, such as radiolabeled amino acids, have improved the detection of metabolically active tumor regions and may help differentiate tumor recurrence from treatment-related changes [26]. Recent evidence suggests that 18-fluoride-fluoro-ethyl-tyrosine positron emission tomography (FET-PET) may improve the diagnostic accuracy to GBM recurrence [27]. Also, fluorescence-guided surgery has emerged as a valuable intraoperative adjunct in GBM surgery. The use of 5-aminolevulinic acid (5-ALA) allows visualization of tumor tissue during surgery, improving the identification of malignant areas and increasing the extent of tumor resection [28].

## Chemotherapy in malignant gliomas: historical milestones

A cornerstone therapeutic option for glioma cure is chemotherapy, which literally means “*chemical therapy*”, is a medical treatment that implies the use of chemical substances, known as chemotherapeutic agents or drugs, to treat diseases, particularly cancer. Chemotherapy aims to inhibit the proliferation of rapidly dividing cells, especially cancer cells, by avoiding their ability to divide and replicate. Although tumor cells are the primary target, chemotherapy may, unfortunately, also affect normal, rapidly proliferating healthy cells, including those in the bone marrow and the gastrointestinal tract lining.

Chemotherapy for malignant gliomas emerged in the mid-20th century. Early agents, such as nitrogen mustards, represented the first successful attempts at systemic chemotherapy [29]. Around 1978, the introduction of alkylating agents (nitrosoureas), such as carmustine (BCNU), provided some therapeutic benefit. The effectiveness of BCNU alone and the combination of BCNU with radiotherapy provided better survival outcomes compared to either treatment alone in anaplastic glioma patients. But the benefits must be balanced with the increased toxicity and side effects, highlighting the need for individualized treatment plans [30].

A significant advance came in the early 1980s with the development of TMZ, an oral alkylating agent derived from dacarbazine synthesized in 1978 at Aston University (UK) as a potential therapeutic agent for melanoma [31]. Building upon the pioneering work conducted at Cancer Research UK, innovative laboratory experiments were performed to create novel drug prototypes and evaluate their efficacy against cancer [32]. Based on the antitumor activity of TMZ [33], its approval by the U.S. Food and Drug Administration (FDA) in 1999 marked a significant advancement in neuro-oncology, as it remains until today the standard of care for GBM patients [34, 35].

A list of FDA-approved agents for the treatment of brain tumor is reported in the review of Qi et al [36]. From 2005, due to its lipophilic nature and ability to cross the blood-brain barrier (BBB), TMZ has been included into GBM therapy, showing an oral bioavailability of 98% [32] and effective penetration into the cerebrospinal fluid, with concentrations approximately 20–30% of those observed in plasma [37, 38]. An important Phase III trial in 2005 demonstrated that combining TMZ with radiation therapy significantly improved survival in patients with newly diagnosed GBM compared with only radiotherapy. This finding led to the establishment of the Stupp Protocol, named after oncologist Roger Stupp, which became the standard treatment for GBM. The trial established a median survival increase from 12.1 to 14.6 months for patients receiving the combined treatment [35]. The standard GBM therapy, which involves a multidisciplinary approach and the Stupp Protocol (endorsed by the EORTC 26981/NCIC CE.3 clinical trial), is widely recognized as an effective regimen. While this multimodal approach has led to moderate improvements in median survival, it underscores the ongoing need for more effective therapies. For this reason, other approaches or drugs were tested and introduced for glioma treatment. From the 1990s, specific genes and molecular pathways regulating cancer growth were discovered and then targeted therapies have emerged as a promising approach to improve treatment outcomes, specifically by targeting molecular alterations in tumor cells. These therapies aim to inhibit key signalling pathways involved in tumor growth and survival, such as the EGFR pathway, often dysregulated in GBM [39]. Studies have explored the efficacy of EGFR activity inhibitors (i.e., erlotinib or gefitinib) in recurrent GBM [40]. However, clinical trial results have demonstrated its inability to consistently improve progression-free survival (PFS) or overall survival (OS) in recurrent gliomas. This limited efficacy may be due to poor penetration of the drug into the tumor tissue or the development of resistance mechanisms [41]. Based on these unsatisfactory results, combinations of erlotinib with other therapies, such as TMZ, have been explored. Although the combination of targeted therapies with TMZ-based chemotherapy and radiotherapy significantly improved OS in a Phase II trial, no such improvement was observed in the Phase III trial. Notably, the incidence of adverse events in the patient group receiving targeted therapy combined with radiotherapy and chemotherapy was not statistically higher compared to those receiving radiotherapy and chemotherapy alone [42]. An open-label, randomized Phase III trial demonstrated that the combination of TMZ with lomustine, another alkylating agent, significantly improves OS compared with standard TMZ therapy in newly diagnosed GBM patients with MGMT promoter methylation, despite an increased risk of hematologic toxicity [43].

Following oral administration, TMZ displays rapid systemic availability, a short plasma half-life of approximately 2 hours, and rapid conversion to the active metabolite 5-(3-methyltriazen-1-yl)-imidazole-4-carboxamide (MTIC), with renal excretion representing the main elimination way [44]. Despite being the FDA-approved chemotherapeutic standard for GBM, TMZ induces clinically significant toxicities, most notably haematological suppression (leukopenia and thrombocytopenia), gastrointestinal disorders, fatigue, and an increased risk of infections and bleeding, hepatic dysfunction, and electrolyte imbalance, which may occur less frequently but become particularly relevant during prolonged therapeutic regimens [45].

The limited clinical efficacy of TMZ can be attributed to the activation of DNA repair mechanisms and adaptive stress responses in surviving tumor cells. Its cytotoxic activity is primarily mediated by DNA alkylation, leading to the formation of multiple methylated DNA lesions. Among these, O6-methylguanine represents the most biologically relevant lesion, as it mispairs with thymine during DNA replication and activates error-prone mismatch repair, ultimately resulting in DNA double-strand breaks and cell death [46]. Although apoptosis is a major outcome of TMZ-induced genotoxic stress, alternative cellular responses, including senescence, may also occur [47, 48]. The efficacy of TMZ is significantly influenced by MGMT, a DNA repair enzyme that removes alkyl adducts from O6-alkylguanine, finally counteracting TMZ-induced DNA damage. In tumors that present an unmethylated MGMT promoter, MGMT expression reduces TMZ sensitivity by repairing O6-methylguanine lesions. On the contrary, promoter hypermethylation suppresses MGMT expression, impairing DNA repair and enhancing TMZ responsiveness. Approximately 50% of GBM patients harbor MGMT-methylated tumors, a feature commonly associated with improved therapeutic response and prognosis [49, 50]. However, TMZ resistance can also arise in MGMT-deficient tumors through MGMT-independent mechanisms, including defects in mismatch repair and activation of alternative DNA repair pathways such as base excision repair and homologous recombination [51, 52]. Consequently, MGMT promoter methylation remains a key predictive biomarker for TMZ response in GBM patients [53, 54]. Despite these therapeutic advances, TMZ remains non-curative and provides only modest survival benefits, underscoring the need for strategies able to overcome intrinsic and adaptive resistance mechanisms [55]. In this context, Tables 1 and 2 summarize preclinical studies (*in vitro* and *in vivo* models) and recent clinical trials investigating the therapeutic potential of TMZ-based combination strategies in GBM.

**Table 1:** Recent preclinical studies (*in* *vitro* and/or *in* *vivo* models) exploring TMZ-based therapies in GBM.

**Study title**	**Description**	**Year**	**Mechanism of action/pathway involved**	**Ref.**
Evaluation of temozolomide and fingolimod treatments in glioblastoma preclinical models	Preclinical study evaluates the effects of TMZ and fingolimod (an immunomodulatory drug) in GBM 2D and 3D models.	2023	Fingolimod inhibits the S1P/S1PR axis, potentially enhancing TMZ efficacy via immune and TME modulation.	[116]
Loco-regional treatment with temozolomide-loaded thermogels prevents glioblastoma recurrences in orthotopic human xenograft models	This study investigated the use of TMZ-loaded thermogels for loco-regional treatment in GBM models, aiming to improve drug delivery and therapeutic outcomes.	2023	The thermogels provide sustained release of TMZ at the tumor site, potentially improving its efficacy while reducing systemic toxicity.	[117]
Efficacy of EGFR plus TNF inhibition in a preclinical model of temozolomide-resistant glioblastoma	Evaluation of the efficacy of combined EGFR and TNF inhibition in an orthotopic murine model of GBM, compared to standard treatment with TMZ.	2019	Dual inhibition reduced tumor-promoting inflammation and EGFR signalling, enhancing antitumor immunity.	[118]
Piperlongumine conquers temozolomide chemoradiotherapy resistance to achieve immune cure in refractory glioblastoma via boosting oxidative stress-inflamation-CD8+-T cell immunity	Piperlongumine enhances chemoradiotherapy efficacy and overcomes resistance in GBM by promoting anti-tumor immunity.	2023	Piperlongumine restores ROS levels reduced by RT/TMZ therapy, activating oxidative stress-related genes and immune responses, while reducing tumor cell proliferation/invasion and increasing apoptosis.	[119]
NEO212, temozolomide conjugated to NEO100, exerts superior therapeutic activity over temozolomide in preclinical chemoradiation models of glioblastoma	The molecule NEO212 (TMZ–NEO100 conjugate) showed stronger anticancer activity than single agents in LN229, T98G, U251, and TMZ-resistant LN229TR2 cells (*in silico* and *in vivo*).	2024	Enhanced DNA damage, reduced MGMT activity; *in silico* modelling showed enhanced drug delivery and efficacy by targeting tumor-specific pathways.	[120]
The Temozolomide–Doxorubicin paradox in Glioblastoma in vitro–*in silico* preclinical drug-screening	Combined *in vitro* and *in silico* screening revealed a paradoxical antagonism between TMZ and doxorubicin in GBM U87MG and primary culture.	2024	*In silico* analysis demonstrates doxorubicin may reduce TMZ cytotoxicity in GBM by enhancing DNA repair and modulating cell cycle progression.	[121]
Patient-derived organoids recapitulate glioma-intrinsic immune program and progenitor populations of glioblastoma	Preclinical study using patient-derived GBM organoids to model tumor-intrinsic immune programs and progenitor cell populations, enabling evaluation of therapeutic responses including TMZ.	2024	Organoids maintain GBM heterogeneity, immune interactions, and stem/progenitor populations, providing a relevant model for testing TMZ efficacy and resistance mechanisms.	[122]
Overexpression of miR-124 enhances the therapeutic benefit of TMZ treatment in the orthotopic GBM mice model by inhibition of DNA damage repair	Preclinical study *in vitro* (GBM cell lines) and *in vivo* (orthotopic GBM mouse model) showing that miR-124 overexpression improves the therapeutic benefit of TMZ.	2025	Inhibition of DNA damage repair via suppression of RAD51, impairing homologous recombination and enhancing TMZ cytotoxicity.	[123]

S1P: sphingosine-1-phosphate; S1PR: sphingosine-1-phosphate receptor; RT: radiotherapy; RAD51: molecule in DNA damage repair.

**Table 2:** Selected clinical trials evaluating TMZ-based therapeutic strategies in GBM patients.

**Trial No./Year/Status/Phase**	**Description**	**Mechanism of action/pathway involved**	**Ref.**
**NCT05977738** **2023** **C** **Phase I**	Trial investigating the safety, tolerability, and pharmacokinetics of pitavastatin (statin) in combination with TMZ in patients with recurrent or progressive GBM.	Pitavastatin inhibits HMG-CoA reductase, potentially disrupting cholesterol synthesis and prenylation pathways critical for cell survival and enhance TMZ sensitivity through redox and metabolic modulation.	[124]
**NCT05629702** **2024** **R** **Phase II**	A multicenter, double-blind, placebo-controlled trial evaluated the efficacy of combining nabiximols (a cannabinoid extract, trade name Sativex^®^) with TMZ in patients with recurrent MGMT-methylated GBM.	Modulation of cannabinoid receptors, potentially affecting tumor growth and chemotherapy response.	[114]
**NCT06095375** **2022** **ANR** **Phase I**	Open-label dose-escalation study assessing the safety and maximum tolerated dose of regorafenib and TMZ, with or without radiotherapy, in newly diagnosed MGMT-methylated, IDH-wild-type GBM patients.	Regorafenib is a multi-kinase inhibitor targeting tumor proliferation and angiogenesis pathways.	ongoing
**NCT04922723** **2022** **ANR** **Phase I/II**	The trial evaluates the safety and efficacy of daratumumab (an anti-CD38 monoclonal antibody) combined with radiation therapy and TMZ in newly diagnosed GBM patients.	Daratumumab targets CD38, which is expressed on tumor cells, enhancing the anti-tumor effects of standard therapies.	ongoing
**NCT00869401** **2025** **C** **Phase I/II**	Randomized placebo-controlled trial of dasatinib with RT/TMZ in newly diagnosed GBM; no survival benefit was observed.	Dasatinib inhibits Src protein-tyrosine kinase to reduce invasion and enhance radiosensitivity with RT/TMZ.	[125]
**NCT03405792** **2025** **ANR** **Phase II**	Evaluated the safety and efficacy of combining TTFields, pembrolizumab (an anti–PD-1 monoclonal antibody), and TMZ in patients with newly diagnosed GBM.	TTFields disrupt cancer cell division, while pembrolizumab exerts immune checkpoint inhibition.	[126]
**CENTRIC EORTC 26071-22072** **2025** **C** **Phase III**	Retrospective analysis of phase III trial assessing the impact of TMZ dosing time (morning vs. evening) on treatment efficacy in newly diagnosed GBM patients.	Chronotherapy approach based on circadian regulation influencing DNA repair and TMZ sensitivity.	[127]
**NCT01765088** **2023** **R** **Phase III**	Compared the efficacy of TMZ combined with interferon alfa versus TMZ alone in patients with newly diagnosed high-grade gliomas. The combination treatment prolonged survival, especially in patients with MGMT unmethylated tumors, with tolerable toxic effects.	Immunomodulation of interferon alfa enhancing antitumor activity.	[128]
**NCT02770378** **2021** **C** **Phase I/II**	Investigated the safety and efficacy of a combination therapy (CUSP9v3) involving nine repurposed drugs alongside continuous low-dose TMZ in patients with recurrent GBM.	Multi-targeted approach aiming to disrupt various survival pathways in GBM cells.	[129, 130]
**NCT03529448** **2023** **ANR** **Phase I/II**	Trial evaluating the safety and tolerability of combining TN-TC11G (a THC/CBD-based therapy) with standard radiotherapy and TMZ in patients with newly diagnosed GBM.	THC/CBD exerts anti-tumor, anti-inflammatory, and pro-apoptotic effects combined with DNA-damaging TMZ and radiation.	ongoing
**NCT05664243** **2023** **ANR** **Phase Ib/II**	Trial assessing the safety and efficacy of DeltEx γδ T cell immunotherapy (allogeneic/autologous) in combination with maintenance TMZ in patients with newly diagnosed or recurrent GBM.	Immunotherapy using engineered γδ T cells to enhance immune-mediated tumor killing combined with TMZ to maintain cytotoxic pressure.	ongoing
**NCT06410248** **2024** **ANR** **Phase I**	Trial evaluating the safety and dosing of Triapine (a ribonucleotide reductase inhibitor) in combination with TMZ in patients with recurrent GBM.	Triapine impairs DNA synthesis and repair, and in combination with TMZ, enhances cytotoxic DNA damage.	ongoing
**NCT01849146** **2025** **C** **Phase I**	Trial testing the safety and maximum tolerated dose of adavosertib combined with RT and TMZ in patients with newly diagnosed or recurrent GBM.	Adavosertib blocks Wee1 kinase, abrogates G2/M checkpoint, and drives RT/TMZ-damaged cells into premature mitosis causing mitotic catastrophe and death.	[131]
**NCT02101905** **2025** **C** **Phase I**	Pre-surgery “pulsatile” lapatinib (tyrosine-kinase inhibitor) in recurrent EGFR-amplified high-grade glioma to assess brain penetration and EGFR inhibition.	Lapatinib blocks EGFR signalling preventing downstream pathways critical for cell survival and proliferation.	/
**NCT05095376** **2022** **R** **Phase III**	Randomized trial evaluating whether the combination of lomustine, TMZ, and radiation improves overall survival in newly diagnosed MGMT-methylated GBM patients.	DNA alkylation and crosslinking (lomustine + TMZ) combined with DNA damage from radiation to enhance cytotoxicity in MGMT-methylated GBM.	[132]

R: recruiting; C: completed; ANR: active, not recruiting; HMG-CoA: 3-hydroxy-3-methyl-glutaryl-coenzyme A reductase; RT: radiotherapy; TTFields: Tumor Treating Fields; THC: tetrahydrocannabinol; CBD: cannabidiol; EGFR: epidermal growth factor receptor.

## The paradox of TMZ in GBM: unravelling its efficacy and potential-unintended effects

Due to the complex and multifactorial mechanisms underlying chemoresistance in GBM, the clinical efficacy of chemotherapeutic agents is significantly limited. In this context, the paradoxical behavior of TMZ is highlighted, underscoring its dual role in GBM therapy. Experimental evidence, primarily derived from *in vitro* studies, indicates that while TMZ effectively induces DNA damage and reduces tumor cell proliferation, it may also promote the emergence of chemoresistant glioma stem cell populations and exert a dose-dependent immunomodulatory effect. At clinically relevant concentrations (≤ 25–50 µM), TMZ has been shown to promote apoptosis and autophagy in GBM cell lines, supporting its cytotoxic activity [56]. However, increasing studies suggest that TMZ can also act as a cytostatic agent by inducing therapy-related senescence. Dose-response experiments in MGMT-deficient GBM cell models demonstrated a linear relationship between TMZ-induced DNA lesions and the induction of apoptosis and senescence, with an evident variability among different cell lines and no apparent threshold effect [48, 57].

**Figure 1:**
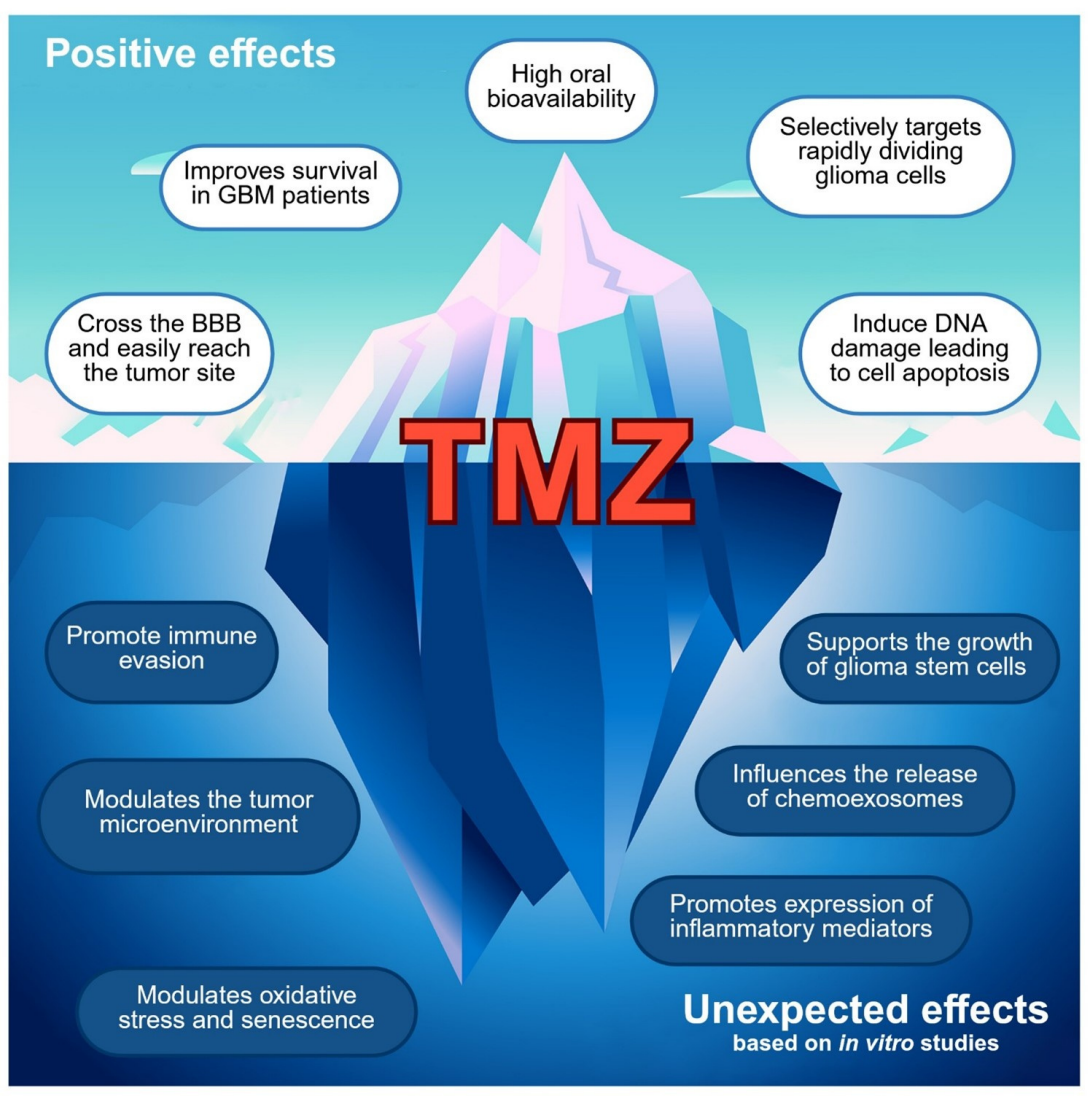
**Effects of TMZ treatment in GBM: promising benefits with unexpected *in* *vitro* findings.** This figure illustrates the dual effects of TMZ treatment in GBM, highlighting both the expected positive outcomes, such as improved survival, BBB penetration, and induction of apoptosis, and the unexpected adverse effects, including immune evasion, modulation of the TME, oxidative stress, and promotion of glioma stem cell growth. These findings are from *in vitro* studies. Palumbo, P. (2026). Created in BioRender. https://BioRender.com/68fxh4s.

Based on the cited *in vitro* studies, Figure 1 aims to represent TMZ as an iceberg, illustrating its paradoxical nature in GBM by dividing its actions into two parts. The visible portion of the iceberg represents the beneficial effects of TMZ supported by clinical evidence, while the hidden portion illustrates unexpected effects mainly described in preclinical studies (*in vitro* and *in vivo*). TMZ is characterized by high oral bioavailability [32, 44], the ability to cross the BBB and efficiently reach the tumor site [32], and a selective targeting of rapidly dividing glioma cells [57–59]. Through the induction of DNA damage, TMZ induces tumor cell apoptosis [57–59], ultimately contributing to improved survival in GBM patients [35]. Conversely, TMZ also exerts a series of unexpected and potentially detrimental effects, mainly emerging from *in vitro* studies. TMZ has been shown to promote immune evasion [60–62] and reshape the TME toward a pro-tumorigenic state [60–64]. In addition, TMZ can sustain the growth and maintenance of glioma stem cells [61, 65–71] and alter the release and molecular cargo of chemoexosomes [64, 72, 73]. These alterations are followed by the upregulation of inflammatory mediators [47, 61, 74–78] and by modulation of oxidative stress responses and therapy-induced senescence [47, 79–88], which may ultimately contribute to treatment resistance and tumor recurrence.

## Beyond chemotherapy: advanced therapeutic approaches in gliomas

Immunotherapy in cancer began to rise in the early 2000s, thanks to the first studies on immune checkpoint inhibitors. In gliomas, the development of immunotherapy has particularly advanced over the past two decades [89]. Several studies were performed using checkpoint inhibitors (targeting immune checkpoints, such as PD-1 and CTLA-4) in GBM, however, the results were inconsistent due to the unique immune environment and the highly immunosuppressive nature of gliomas [90].

In 2009 the FDA approved the use of Bevacizumab, an anti-vascular endothelial growth factor (VEGF) monoclonal antibody, for recurrent GBM [91]. It marked the first targeted therapy approved for brain tumors, aiming to inhibit tumor angiogenesis.

The Tumor Treating Fields (TTFields) is a non-invasive cancer treatment that uses low intensity alternating electric fields to disrupt cancer cells’ division, inducing cancer cell death. In 2011, the FDA approved TTFields as a treatment for recurrent GBM. These fields are generated by adhesive transducer arrays applied to the patient’s scalp, which produce electric fields at specific frequencies. The significance of TTFields increased following the EF-14 Phase III trial, which demonstrated that combining TTFields with TMZ notably enhanced PFS and OS in patients with newly diagnosed GBM [92].

The heterogeneity of gliomas necessitates personalized treatment strategies. Molecular profiling of tumors allows for tailored therapies targeting specific genetic alterations. In this scenario, one of the most recent, promising and rapidly evolving fields in the personalized treatment of malignant brain tumors is the gene therapy. This strategy has the potential to transform therapeutic approaches for GBM by offering targeted treatments that overcome the limitations of conventional therapies like surgery, chemotherapy, and radiation, which often fail to address the underlying genetic abnormalities of the tumor. Gene therapy targets the genetic alterations within tumor cells by introducing therapeutic genes or inactivating oncogenes that drive tumor growth. This approach aims to halt tumor progression while enhancing the immune system's response, offering a more precise and effective treatment for glioma compared to traditional methods [93, 94].

GBM is characterized by various genetic mutations, including alterations in oncogenes and tumor suppressor genes, such as mutations in TP53, amplification of EGFR, and loss of function in phosphatase and tensin homolog (PTEN). These mutations contribute to the aggressive nature of GBM and its resistance to standard treatments. Several gene therapy procedures have been explored for GBM treatment, including suicide gene therapy, where tumor cells are made sensitive to specific drugs, oncolytic viral therapy, which uses viruses engineered to infect and destroy cancer cells, cytokine-mediated gene therapy, and tumor suppressor gene restoration and CRISPR/Cas9 gene editing [95, 96]. These approaches, when combined with traditional therapies, have shown the potential to improve treatment outcomes by synergistically increasing tumor cell death and minimizing the toxicity associated with conventional methods. One of the main advantages of gene therapy is its ability to personalize treatments based on the genetic profile of an individual’s tumor. For instance, oncolytic viruses can be engineered to selectively infect GBM cells that express particular molecular markers, leaving healthy brain tissue unharmed and reducing side effects. Additionally, gene therapy can bypass the challenges of the BBB by delivering viral vectors directly into the tumor or surrounding brain tissue, ensuring that therapeutic genes or oncolytic viruses reach the tumor site. An exciting development in this field is the enhancement of the body’s immune response through gene therapy. Several gene therapy approaches have explored the targeting of growth factor signalling pathways, such as TGF-β, EGFR and the IGF axis, which play important roles in GBM progression and TME interactions [97–100]. Approaches like CAR T-cell therapy, where T-cells are genetically modified to express receptors targeting GBM cells, have shown promising results in early clinical trials [101]. Combining standard approaches with gene therapy could synergistically increase GBM cell death, overcome GBM’s resistance to traditional approaches, and reduce the toxicity of conventional treatments.

**Figure 2:**
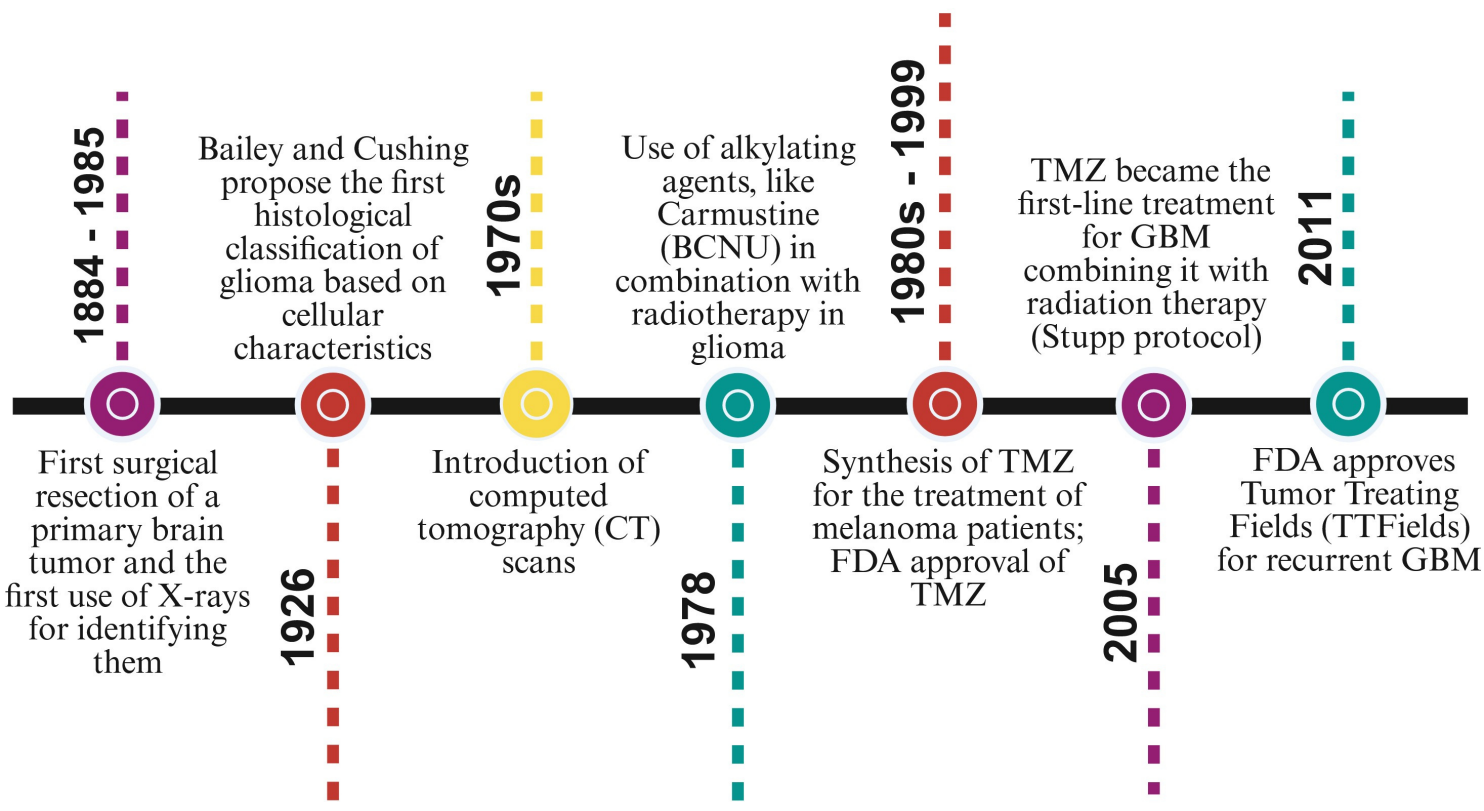
**Timeline of key milestones in brain tumor therapy.** The figure presents major advances in GBM diagnosis and treatment, from early surgical resections and imaging technologies to chemotherapy, radiotherapy optimization, and the introduction of Tumor Treating Fields (TTFields). Palumbo, P. (2026). Created in BioRender. https://BioRender.com/vkap339.

Overall, the treatment of malignant gliomas has evolved through gradual and significant advancements. From early, basic surgical methods to today’s sophisticated multimodal therapies, each development has deepened the understanding of these complex tumors. A timeline highlighting the crucial developments in brain tumor therapy approaches is presented in Figure 2.

## From chemotherapy to precision medicine in GBM

In GBM, precision medicine involves tailoring therapeutic strategies based on comprehensive molecular profiling, combining tumor-specific molecular features with clinical and biological patient characteristics. Precision medicine relies on genomic, transcriptomic, and proteomic analyses to identify specific genetic mutations, dysregulated molecular pathways associated with poor prognosis in GBM, and sets of genes that are overexpressed and drive tumor growth, protein expression, and post-translational modifications [102–104]. Notably, precision medicine in GBM does not imply the identification of a single oncogenic driver, but rather complex, biologically informed stratification of patients based on comprehensive molecular and omics profiles with the aim of predicting therapeutic response, resistance, and adaptive tumor behavior. Recent reviews highlight that the heterogeneous molecular status of GBM requires stratification using integrated genomic, transcriptomic, and proteomic data rather than targeting a single oncogenic driver [105, 106].

In this context, in 2017, the European Society for Medical Oncology (ESMO) published an ESMO Precision Medicine Glossary in which it was clarified that the terms “precision medicine” and “personalized medicine” are technically interchangeable, however, the term “precision medicine” is preferred to avoid the misleading idea of treatments being unique for each individual, and to better reflect the highly accurate nature of new genomic technologies [107]. Precision medicine has also driven the development of innovative clinical trial designs, including basket, umbrella, and adaptive trials, which enable efficient evaluation of multiple targeted or biomarker-driven therapeutic strategies within molecularly defined patient subgroups [108–110].

Here, we summarize selected completed and ongoing clinical trials that exemplify the application of precision medicine approaches in glioma and GBM.

The INDIGO trial (NCT04164901), a randomised, double-blind, placebo-controlled trial, is one of the first in glioma with a precision medicine approach, targeting mutations in the gene encoding IDH1, with the brain-penetrant mutant IDH inhibitor, vorasidenib. Results show that vorasidenib significantly reduced tumour growth rate and improved seizure control compared with placebo, without detrimental effects on health-related quality of life or neurocognitive function. Additional follow-up confirmed the PFS and time to next intervention in patients with grade 2 IDH1/2-mutant diffuse glioma [111].

A key example of an umbrella precision medicine trial in GBM is the Neuro Master Match (N2M2) study (Phase I/II) (NCT03158389), which applies comprehensive tumor molecular profiling, including whole-methylome analysis, whole-exome sequencing, whole-genome sequencing, RNA sequencing, and immunohistochemistry, in patients with newly diagnosed GBM and an unmethylated MGMT promoter. Patients with “actionable” alterations were evaluated by a molecular tumor board and assigned to substudies involving radiation with concurrent and adjuvant palbociclib (CDK4/6 inhibitor), temsirolimus (mTORC1 inhibitor), idasanutlin (MDM2 inhibitor), alectinib (Alk inhibitor), or vismodegib (Smoothened inhibitor). Patients lacking actionable alterations were randomized to therapy with atezolizumab (anti-PD-L1 antibody), asunercept (FAS-ligand inhibitor), or TMZ (standard chemotherapy) during and after radiation. Relevant results included limited to no enrolment in the vismodegib, alectinib, and idasanutlin substudies; no therapeutic benefit with palbociclib, atezolizumab, or asunercept; and, in contrast, promising PFS and OS with temsirolimus [112]. Overall, the N2M2 trial demonstrated how an umbrella trial approach can efficiently evaluate multiple clinical regimens and identify those regimens with limited clinical potential. Compared with traditional Phase II testing, which would have required separate trials enrolling at least 150 patients per regimen, the N2M2 study evaluated seven investigational agents and identified temsirolimus as the only promising treatment, with a total enrollment of 301 patients [112].

GBM Adaptive, Global, Innovative Learning Environment (GBM AGILE) (NCT03970447) is an international, seamless Phase II/III response-adaptive platform study designed to evaluate multiple therapeutic agents at the same time in GBM. The design of this trial is based on Bayesian response-adaptive randomization and matches effective therapies with patient subtypes. Continuous learning within the clinical setting is possible through dynamic treatment matching to emerging efficacy signals across molecularly defined patient subsets, making this trial an example of how to leverage adaptive trial designs to advance precision medicine strategies in GBM [113].

The ARISTOCRAT trial (NCT05629702) is a Phase II, multi-centre, double-blind, placebo-controlled, randomised study assessing the addition of cannabinoids (nabiximols) in patients with recurrent MGMT methylated GBM who are suitable for treatment with TMZ. By selectively enrolling MGMT-methylated tumors, the study exemplifies a biomarker-driven precision medicine approach to optimize therapeutic efficacy within a molecularly defined patient subgroup. After completing first-line therapy, patients who have relapsed for at least three months are randomly assigned to either nabiximols or a placebo in combination with TMZ in a 2:1 ratio. The primary achievement is OS time, defined as the total number of days from randomisation to death. Secondary endpoints include OS at 12 months, as well as PFS time, health-related quality of life (HRQoL), and treatment-related adverse events. The ARISTOCRAT study incorporates modulation of tumor biology and the TME as a precision strategy instead of relying solely on biomarker stratification, to explore whether if cannabinoid-mediated effects on inflammation, stress responses, and tumor-host interactions can improve TMZ effectiveness and clinical outcomes in a specific GBM population [114, 115].

The PEAR-GLIO (NCT06038760) trial introduces an AI-driven *ex vivo* precision medicine platform that uses patient-derived 3D immune-microtumor models to assess therapeutic sensitivity to approved and experimental agents. PEAR-GLIO is a UK-based, observational study enrolling 50 patients diagnosed with operable primary brain tumors, including grades II–IV gliomas. Tumor-derived and immune patient cells are cultured as physiologically relevant 3D immune-microtumors and treated with FDA-approved and experimental treatments. Phenotypic and molecular responses, including changes in tumour viability, cell death, migration, and immune cell infiltration, are performed using live imaging and computer vision. *Ex vivo* drug responses, assessed *in vivo* confocal imaging with multiomic profiling, link treatments performed at the functional level to established molecular biomarkers such as MGMT promoter methylation, IDH mutation status, and 1p/19q co-deletion in the study. The first aim of this study is to establish an *ex vivo* model and confirm whether approved therapies exhibit their intended mechanism of action in the model. Secondary objectives include correlation of *ex vivo* results with clinical outcomes, if available.

An additional precision-oriented trials ongoing included in the UK next-Generation aGile Genomically Guided Glioma platform (5G) and are the 5G-EMERALD and 5G-RUBY trials, which illustrate complementary pathway-directed precision medicine strategies in IV grade malignant glioma.

In particular, the 5G-EMERALD study (NCT06632236) is a Phase I trial evaluating amivantamab, a bispecific monoclonal antibody uses to target EGFR and MET (a tyrosine kinase receptor), in patients with high-grade malignant brain tumors. The rationale of this trial is based on the frequent dysregulation of EGFR and MET signalling pathways in GBM and their role in progression and therapeutic resistance [97, 111]. In this trial, molecular alterations in the EGFR and MET signalling pathways are systematically analyzed to understand which tumors are more responsive to amivantamab and to explore the mechanisms that may underlie treatment resistance. Instead of relying on molecular features as strict eligibility criteria, this biologically informed precision strategy uses molecular data to interpret treatment outcomes and help identify pathway-dependent patient subgroups that are relevant to future therapeutic stratification.

Moreover, the 5G-RUBY trial (NCT06630260) uses a precision medicine-based patient selection strategy, enrolling patients with GBM whose tumors are molecularly characterized by NF1 loss or activation of the RAS–RAF–MEK signalling pathway. In this study, the combination of avutometinib, a RAF/MEK pathway inhibitor, and defactinib, a focal adhesion kinase (FAK) inhibitor, is used to target both oncogenic signalling pathways and TME interactions in patients.

## Conclusion

Therapeutic strategies for GBM have progressively evolved from basic surgical approaches to more sophisticated, molecularly informed strategies. Although the discovery and the widespread use of TMZ represented a significant advance in the treatment of GBM patients, its limited efficacy and the development of adaptive cellular resistance mechanisms highlight the urgent need for novel therapeutic strategies beyond conventional chemotherapy.

In this context, precision medicine approaches play a central role, particularly in GBM, a tumor characterized by marked intratumoral and intertumoral heterogeneity. By integrating biomarker-driven patient stratification, pathway-informed therapeutic strategies, and adaptive clinical trial designs, precision medicine provides a rational and adaptable tool to guide treatment selection, enabling the identification of context-dependent features and offering new opportunities to counteract therapeutic resistance and tumor recurrence. Collectively, precision medicine underscores the necessity for tailored therapeutic approaches capable of recapitulating the physiological complexity of GBM, ultimately paving the way toward more effective and durable clinical outcomes.

## Abbreviations

Alk: anaplastic lymphoma kinase
BBB: blood-brain barrier
BCNU: carmustine (1,3-bis(2-chloroethyl)-1-nitrosourea)
CDK4/6: cyclin-dependent kinases 4 and 6
CT: computed tomography
CTLA-4: cytotoxic T-lymphocyte antigen 4
EGFR: epidermal growth factor receptor
ESMO: European Society for Medical Oncology
FDA: Food and Drug Administration
GBM: glioblastoma
IDH: isocitrate dehydrogenase
IGF: insulin-like growth factor
MDM2: mouse double minute 2
MGMT: O6-methylguanine-DNA methyltransferase
MRI: Magnetic Resonance Imaging
mTORC1: mechanistic target of rapamycin complex 1
OS: overall survival
PD-1: programmed cell death protein 1
PD-L1: programmed death-ligand 1
PET: positron emission tomography
PFS: progression-free survival
TME: tumor microenvironment
TMZ: temozolomide
TTFields: Tumor Treating Fields
WHO: World Health Organization

## Declarations

### Copyright

© The Author(s) 2026.
